# Paradoxical phenomena of bullous pemphigoid induced and treated by identical biologics

**DOI:** 10.3389/fimmu.2022.1050373

**Published:** 2023-01-05

**Authors:** Jie Zhang, Si-Hang Wang, Ya-Gang Zuo

**Affiliations:** Department of Dermatology, State Key Laboratory of Complex Severe and Rare Diseases, Peking Union Medical College Hospital, Chinese Academy of Medical Sciences and Peking Union Medical College, National Clinical Research Center for Dermatologic and Immunologic Diseases, Beijing, China

**Keywords:** bullous pemphigoid, TNF-α inhibitor, IL-17 inhibitor, IL12/IL23 inhibitor, paradoxical phenomena

## Abstract

**Objective:**

This study aimed to investigate the clinical features of biologics-induced bullous pemphigoid (BP) and the therapeutic effects of those agents for BP, exploring the underlying pathophysiological mechanisms.

**Methods:**

We searched PubMed, Web of Science, and Elsevier for studies involving pemphigoid patients treated with or induced by identical biologics published in English from January 2009 to April 2022.

**Results:**

Seventeen cases of drug-induced BP associated with anti-tumor necrosis factor (aTNF)-α therapies, one with interleukin (IL)-17 inhibitors, and seven with IL-12/IL-23 or IL-23 inhibitors were enrolled. Time to cutaneous toxicity varied among different types of agents, and the characteristics of clinical examinations were similar to idiopathic BP. Discontinuation of the culprit drugs and initiation of topical or systemic corticosteroids were adequate in most cases. Several monoclonal antibodies above have also been reported for the treatment of refractory or recurrent BP, especially concurrent with psoriasis.

**Conclusion:**

Biologics for immune-related diseases, including TNF-α, IL-17, and IL-12/IL-23 or IL-23 inhibitors, can both induce and treat BP, which might be associated with a helper T cells Th1/Th2 imbalance, complicated inflammatory networks, and a specific individual microenvironment, suggestive of a new perspective on the therapeutic algorithms of BP. There have been numerous reports about biologics inducing or treating BP. We have taken note of this phenomenon and focused on biologics with both pathogenetic and therapeutic effects on BP. Our review summarized the clinical characteristics of associated cases, trying to figure out the underlying mechanisms of this paradoxical phenomenon and to provide an integrated perspective and new therapeutic alternatives for BP.

## Introduction

Bullous pemphigoid (BP) is a common autoimmune blistering disease characterized by intense bullae on normal or erythematous skin with prominent pruritus, subepidermal blisters on histological examination, and immunopathological findings showing immunoglobulin (Ig) G and complement (C)3 deposition at the basement membrane zone (BMZ). Autoantibodies targeting BP180 and BP230 lead to the degradation of the BMZ and bulla formation, which can be detected in the sera of most patients (1).

The pathogenesis of BP is quite unclarified, and lots of triggering and predisposing factors, such as central neurological disorders, old age, infections, and medications, were reported to be associated with its occurrence. Administration of specific topical or systemic drugs can be identified as a causal factor of BP (2). Noteworthy, with biologics increasingly utilized in treating a broader spectrum of immune-related diseases, such as psoriasis, rheumatoid arthritis, and inflammatory bowel diseases, biologics-induced BP has been reported more and was highlighted in our review. In addition to BP, other autoimmune blistering diseases (AIBDs), such as pemphigus, pemphigoid nodularis, and linear IgA bullous dermatosis, induced by biologics have also been reported. There may be an underlying etiologic relationship between biological agents and the onset of AIBDs.

Dramatically, biologics-induced BP has also shown an unexpected treatment effect in some reports. Programmed cell death protein-1 (PD-1)/programmed death ligand-1 (PD-L1) inhibitors, the mainstay of biologics causing BP, have been reported in many cases and reviews (3, 4), but to our knowledge, there were no reports to illustrate their roles in the treatment of BP. Hence, in this paradoxical phenomenon, we mainly focused on biologics including tumor necrosis factor (TNF)-α, interleukin (IL)-17, and IL-12/IL-23 or IL-23 inhibitors that have effects of both inducing and treating BP.

## Tumor Necrosis Factor-α blockers

### TNF-α blockers inducing BP

TNF-α blockers, or anti-TNF-α (aTNF-α), have been involved in the treatment of psoriasis vulgaris, rheumatoid arthritis, and inflammatory bowel disease and were recently approved for hidradenitis suppurativa. In the past decades, a growing number of cases of adverse effects, especially autoimmune diseases, have been reported, such as cutaneous vasculitis, lupus-like syndrome, systemic lupus erythematosus, and BP (5, 6). Reviewing the published literature from January 2009 to April 2022, we collected 17 cases (7–23) of aTNF-α-induced BP and analyzed the clinical characteristics (**Tables 1**, **2**).

**Table 1:** Clinical characteristics of BP cases associated with TNF-α inhibitors.

Ref	Age/sex	Primary diagnosis	Past history	TNF-α inhibitors	The total dose (mg)	Time to cutaneous toxicity (weeks)	BP occurring site	Anti-BP180/BP230	BP therapy	Outcome
(7)	F/54	Rheumatoid arthritis	Blood pressure, tuberculous pleuritis	Infliximab	720	12	From the lower abdomen to the groin	ND	Discontinuation of infliximab without initiation of any other drugs	CR
(8)	F/42	Crohn’s disease	Erythema multiforme	Infliximab	900	6	ND	Positive for anti-BP180	Intravenous corticosteroids	CR
(9)	F/54	Ulcerative colitis	ND	Infliximab	600	5	Trunk and limbs	ND	Systemic corticosteroids	CR
(10)	F/69	Ulcerative colitis	ND	Infliximab	900	9	Trunk and extremities	Anti-BP180: 651.2 U/ml	Oral prednisolone	CR
(11)	M/50	Psoriasis vulgaris, psoriatic arthritis	No	Adalimumab	240	12	Trunk and limbs	ND	Oral prednisolone and topical clobetasol	CR but relapse after withdrawal of prednisolone
(12)	F/65	Rheumatoid arthritis	No	Adalimumab	1,040	52	Knees, wrists, elbows and back	ND	Oral prednisolone	CR
(13)	M/49	Ulcerative colitis	Sclerosing cholangitis	Adalimumab	ND	78	Forehead, trunk, scalp, hands, and feet	Anti-BP 180: 126.3 U/ml	Oral prednisone (no response), steroid wet dressings, azathioprine, and IVIG	CR
(14)	M/81	Ulcerative colitis	ND	Adalimumab	ND	18	Trunk and extremities	Positive for anti-BP180	Oral prednisolone, methotrexate	CR
(15)	M/45	Plaque psoriasis	No	Adalimumab	160	4	Trunk and extremities	ND	Oral prednisone	CR but relapse after restarting adalimumab
(16)	F/65	Rheumatoid arthritis	Sjögren’s syndrome	Adalimumab	ND	ND	ND	Anti-BP 180: 22.7 U/ml	Topical glucocorticoid and oral doxycycline	CR but relapse after initiation of tocilizumab
(17)	M/20	Crohn’s disease	No	Adalimumab	2,080	52	Extremities	Positive for anti-BP180	Oral prednisolone and topical clobetasol	CR but relapse when prednisolone was tapering
(18)	M/42	Hidradenitis suppurativa	Renal failure	Adalimumab	ND	48	Forearms, hands and feet	Anti-BP 180: 24 U/ml	Oral prednisolone	CR but relapse after withdrawal of prednisolone
(19)	M/62	Psoriasis vulgaris	Type 2 diabetes mellitus	Adalimumab	4,180	209	Trunk and extremities	Anti-BP 180: 355 U/ml	Oral prednisolone	PR (CR after linagliptin was withdrawn)
(20)	F/71	Rheumatoid arthritis	ND	Adalimumab	3,120	156	Back, neckline, flexor forearms and lower legs	Positive for anti-BP180	Topical corticosteroids	CR
(21)	F/65	Rheumatoid arthritis	ND	Etanercept	1,300	104	Trunk and limbs	Anti-BP 180: 37.28 U/ml	Oral corticosteroid and methotrexate	CR
(22)	F/63	Psoriasis, psoriatic arthritis	No	Etanercept	125	9	Arms and upper back	ND	Topical corticosteroids (unresponsive), then adalimumab (for psoriasis) was dramatically effective	CR
(23)	F/79	Psoriasis	Type 2 diabetes mellitus	Etanercept	25	1	Generalized	ND	Oral dapsone	CR

BP, bullous pemphigoid; TNF-α, tumor necrosis factor α; Ref, reference; CR, complete remission; PR, partial response; ND, not described; F, female; M, male.

**Table 2:** Statistics of BP cases induced and treated by biologics.

Types of biological agents	Biologics	BP cases induced by biologics		BP cases treated by biologics	
		Numbers	Proportion	Numbers	Proportion
TNF-α inhibitors	Infliximab	4	23.53%	−	−
	Adalimumab	10	58.82%	−	−
	Etanercept	3	17.65%	3	100.00%
	Total	17	100.00%	3	100.00%
IL-12/IL-23 or IL-23 inhibitors	Ustekinumab	6	85.71%	1	100.00%
	Guselkumab	1	14.29%	−	−
	Total	7	100.00%	1	100.00%
IL-17 inhibitors	Secukinumab	1	100.00%	2	66.67%
	Ixekizumab	−	−	1	33.33%
	Total	1	100.00%	3	100.00%
**Total**		25		7	

BP, bullous pemphigoid; −: no such cases in our review.

#### Demographic characteristics

Of the 17 cases, 10 women (58.8%) and seven men (41.2%), only one was a 20-year-old young man (17), and the rest were middle-aged and elderly people, who had almost comparable spontaneous BP. The median age of the patients was 57 (mean = 57; range 20–81) years. The primary diseases consisted of five cases of rheumatoid arthritis (29.4%), two cases of Crohn’s disease (11.8%), four cases of ulcerative colitis (23.5%), five cases of psoriasis including psoriatic arthritis (29.4%), and one case of hidradenitis suppurativa (5.9%). Four cases were treated with infliximab (23.5%), 10 with adalimumab (58.8%), and three with etanercept (17.6%) (**Table 1**).

Sixteen (94.1%) of the 17 patients developed blistering lesions in their entire clinical courses. The duration between the initiation of aTNF-α therapy and the onset of BP was variable. After excluding two unspecified cases, the median time from aTNF-α treatment to the onset of BP was 33 weeks (mean = 54 weeks; range 1–209 weeks). One patient, who developed BP within just 3 days of receiving etanercept (23), had the shortest time to bulla on our record. Most of the occurring locations were the trunks and extremities, while only in one case (23) was the initial BP localized on the perianal, perineal, and perivaginal areas. Only two (11.8%) subjects had BP with oral mucosal involvement.

#### Laboratory examinations

Skin biopsies were taken in 12 of the 17 cases for histological examination. Nine patients (75%, 9/12) presented with subepidermal blisters or clefts and three (25%, 3/12) with only inflammatory cell infiltration. A classification of cell infiltration has been described in 11 of the 12 cases. It was manifested in nine cases (81.8%, 9/11) as eosinophil infiltration, three (27.3%, 3/11) as lymphocyte infiltration, and one (9.1%, 1/11) as neutrophil infiltration.

Direct immunofluorescence (DIF) was performed in 12 of the 17 cases, of which 11 (91.7%, 11/12) cases were positive for linear deposition of IgG at the BMZ, 10 (83.3%,10/12) for C3, two (16.7%, 2/12) for IgA and IgM, respectively. Indirect immunofluorescence (IIF) using salt-split normal human skin as a substrate was detected in five patients. Four cases were positive for IgG deposits on the epidermal side, one of which demonstrated C3 deposition as well, and one patient was negative.

Ten (58.8%) of the 17 cases detailed the serum autoantibodies as positive. Apart from one case with unspecified types of BP antibodies, the remaining nine cases were positive for anti-BP180 at different concentrations.

#### Therapy and outcome

Fifteen of the 17 cases were treated with topical and/or systemic corticosteroids (**Table 1**). Systemic corticosteroids were applied in 12 cases intravenously or orally and were efficacious in most cases. Of these 12 cases, one patient (19), whose BP was induced by adalimumab and linagliptin, was in partial response with prednisolone but in complete remission after withdrawing linagliptin. In two cases, corticosteroids were combined with methotrexate for the treatment of BP due to a poor effect of the original medication in one patient.

Six (35.3%, 6/17) cases relapsed, and the others were in complete clinical remission. The cessation and tapering of corticosteroids induced recurrences in four cases, and BP was controlled with an increased dose of prednisolone. A reoccurrence in one patient was due to reintroducing adalimumab, and symptoms were relieved after ceasing the drug (15). In one case, the initiation of tocilizumab for rheumatoid arthritis induced a recurrence of BP, and switching from topical corticosteroids to oral achieved remission of the disease successfully (16).

### TNF-α blockers treating BP

Little information is known regarding the therapeutic use of aTNF-α agents for BP. To our knowledge, there were three reported cases (24–26) of TNF-α antagonists (etanercept) successfully treating BP concomitant with psoriasis (**Tables 2**, **3**).

**Table 3:** Clinical characteristics of BP cases treated by biologics.

Ref.	Age/sex	PASI	Medication history	Biologics treatment	Dose	Course of treatment	Anti-BP180/BP230	EOS	Outcome of BP
(24)	57/M	ND	Cyclosporin, topical corticosteroids and calcipotriol	Etanercept	50 mg qw	6 months	Anti-BP180: 175 U/ml	ND	CR
(25)	49/F	17.2	Cyclosporine, methotrexate and NB/UVB phototherapy	Etanercept	100 mg qw	12 weeks	ND	ND	CR
(26)	64/M	12	Psoralen, ultraviolet A light therapy and topical corticosteroids	Etanercept	50 mg qw then 50 mg biw	ND	ND	ND	CR
(27)	88/F	6.4	Acitretin	Ustekinumab	45 mg at week 0 and week 4, then every 12 weeks	48 months	Anti-BP180: 17 U/ml and anti-BP230: 103 U/ml	700/µl	CR
(28)	37/F	11.6	Topical corticosteroids	Ixekizumab	160 mg at week 0, then 80 mg at weeks 2, 4, 6, 8, 10, and 12, then 80 mg every 4 weeks	1 year	Anti-BP180: 21 U/ml and anti-BP230: 52 U/ml	ND	CR
(29)	68/M	48.5	Intermittent courses of methotrexate, acitretin, cyclosporin, phototherapy and topical therapies	Secukinumab	300 mg at weeks 1, 2, 3, and 4 and then 300 mg every 4 weeks	7 months	ND	400/µl	CR
(30)	85/F	No history of psoriasis	ND	Secukinumab	300 mg at days 1, 8, 15, 22, 50, and 83	12 weeks	Anti-BP180: 689 U/ml	ND	CR

BP, bullous pemphigoid; Ref, reference; PASI, Psoriasis area and severity index; EOS, eosinophil; qw, once a week; biw, twice a week; CR, complete remission; ND, not described; F, female; M, male.

Etanercept was started due to the poor effect of the original drugs (steroids or immunosuppressants), a worry about the potential relapse of psoriasis or converting to pustular psoriasis, and the recurrence or worsening of BP when corticosteroids were tapered. In those cases, all showed significant efficacy for both BP and psoriasis. Coincidentally, in one of our cases, BP triggered by etanercept was unresponsive to topical corticosteroids but settled dramatically after adalimumab administration (22). Interestingly, her psoriasis continued to deteriorate after the switch. Apart from BP, TNF-α inhibitors were found to be effective in the treatment of mucous membrane pemphigoid (31–33) and anti-laminin gamma-1 pemphigoid (34).

### Pathogenesis of TNF-α blockers inducing or treating BP

**Figure 1:**
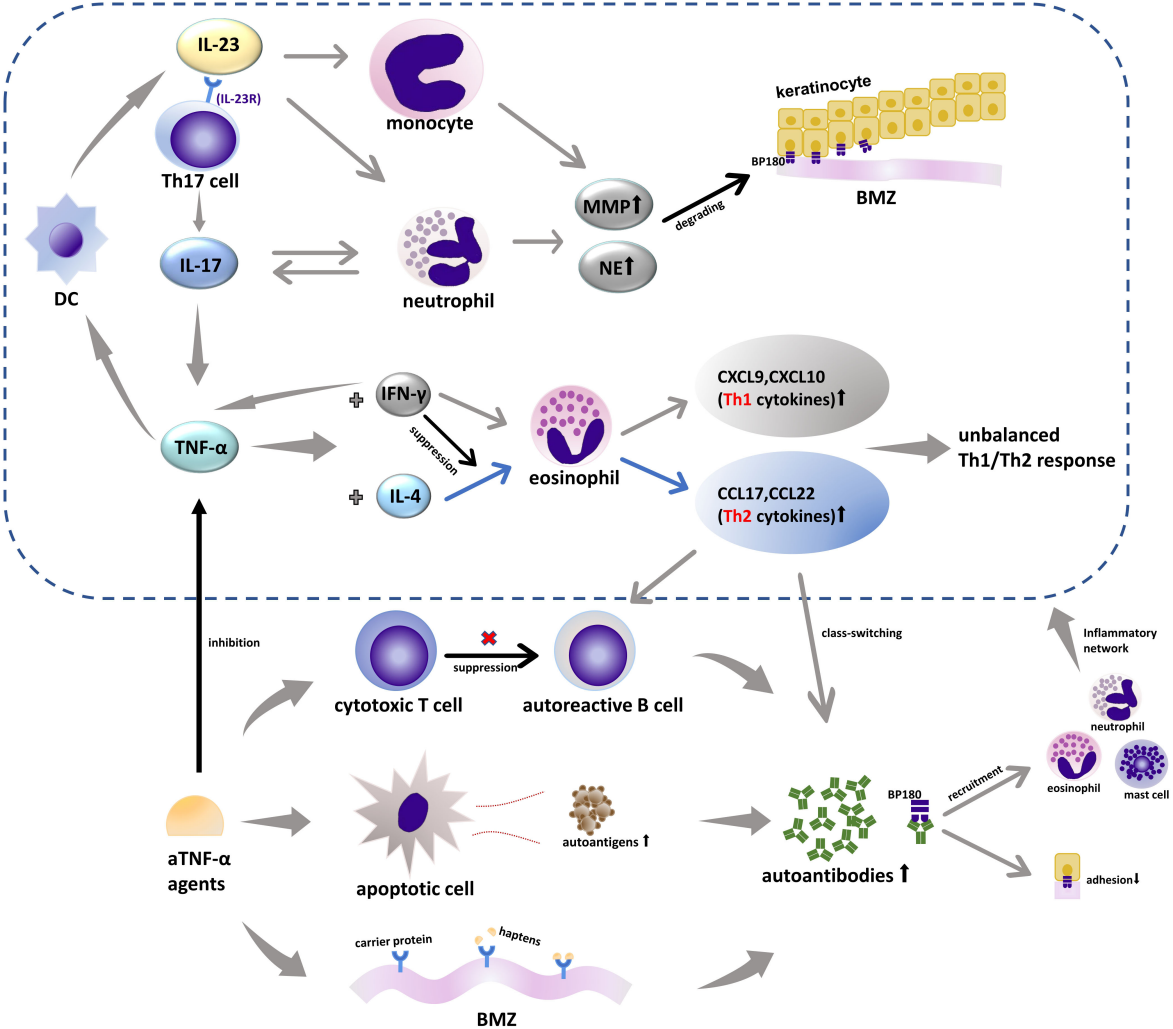
TNF-α-, IL-17-, and IL-23-associated inflammatory networks in BP and biologics. IL-23 acts as an upstream regulatory cytokine for the secretion of IL-17 from Th17 through IL-23R and can induce MMP-9 expression by monocytes alone. IL-17 can promote the production of a series of inflammatory molecules including TNF-α and increase the secretion of MMP-9 and NE from neutrophils that are involved in the formation of subepidermal blisters. TNF-α, which is considered as an upstream product of IL-23, can act on DCs that can secrete IL-23. TNF-α can also induce different immune responses dominated by Th1 or Th2 depending on the micro immune profile (the levels of IL-4 and IFN-γ). IFN-γ reduces the response of eosinophil to IL-4 plus TNF-α and can increase the production of endogenous TNF-α of different cell types in a specific microenvironment. aTNF-α agents increase the production of autoantibodies in three ways: increasing cell apoptosis and espousing autoantigens, inhibiting the cytotoxic T-cell response that was involved in suppressing autoreactive B cells, and acting as antigenic haptens to bind or modify proteins in the BMZ. *TNF-α*, tumor necrosis factor α; *IL-17*, interleukin 17; *IL-23*, interleukin 23; *IL-23R*, interleukin 23 receptor; *MMP-9*, matrix metalloproteinase 9; *NE*, neutrophil elastase; *DC*, dendritic cell; *IL-4*, interleukin 4; Th, helper T cells; *IFN-γ*, interferon γ; *BMZ*, basement membrane zone; *CCL*, chemokine (C-C motif) ligand; *CXCL*, C-X-C motif ligand.

The pathogenesis of BP induced by TNF-α blockers remains unclear and is considered to be linked to the interaction of various immune cascades. Three hypotheses have been put forward to explain the underlying mechanism. Firstly, increased cell apoptosis was found in patients receiving aTNF-α therapies due to which the autoantigen exposure increased and subsequently led to the formation of autoantibodies (35), consistent with one case in our records that reported coexisting anti-desmoglein (Dsg)3 and anti-BP180 autoantibodies (10). Secondly, these individuals have an unbalanced cytotoxic T-cell response, which relieves the suppression of autoresponsive B cells and results in increased autoantibody production (5, 35, 36). Thirdly, TNF-α blockers may have the ability to act as antigenic haptens to bind and modify protein molecules in the BMZ and make them susceptible to an immune attack (37). Autoantibodies targeting BP180 can lead to a decreased adhesion of BP180 and recruitment of inflammatory cells, including mast cells, eosinophils, and neutrophils, activating the inflammatory network consistent with various cytokines and proteases (38). This cascade, in turn, increases the production of TNF-α (**Figure 1**).

TNF-α antagonists can also treat BP, and this paradoxical phenomenon has been noticed. Increased levels of TNF-α have been identified in the serum and blister fluid of BP patients (39, 40), and the serum levels of TNF-α are correlated with BP severity (41). TNF-α is pivotal in the secretion of Th1 [C-X-C motif ligand (CXCL)9, CXCL10]- and Th2 [chemokine (C-C motif) ligand (CCL)17, CCL22]-associated cytokines, which depends on the individual microenvironment, more specifically the level of IL-4 and interferon (IFN)-γ (2, 37, 42). The process of secreting CCL12 and CCL22 is regulated by IL-4 and significantly enhanced in combination with TNF-α, which is associated with a Th2-type response, whereas TNF-α and IFN-γ have a synergistic effect on the generation of CXCL9, CXCL10, and Th1 cytokines. Th2 cells and relevant cytokines can promote B cell proliferation and differentiation into autoreactive ones and the class-switching of autoantibodies, which is considered to be a dominant immune type in the development of BP (43, 44). Various inflammatory factors in the complicated network interact with each other, and individuals in different immune microenvironments will show different immune effects (**Figure 1**). Therefore, we postulate that aTNF-α can both treat Th1-driven diseases such as psoriasis and induce Th2-driven diseases like BP depending on the micro immune profile (the levels of IL-4 and IFN-γ). Namely, TNF-α blockers may potentially suppress the Th2 response, thus becoming an alternative therapy for BP, especially when accompanied by psoriasis.

## Interleukin-12/Interleukin-23 or Interleukin-23 inhibitors

### IL-12/IL-23 or IL-23 inhibitors inducing BP

Anti-IL-12/IL-23 or IL-23 therapies, inhibiting the activity of IL-23 and IL-12, have been approved for the treatment of psoriasis and Crohn’s disease. Observed adverse effects regarding dermatological manifestations include atopic dermatitis, cutaneous lupus erythematosus, BP, and so on. Collecting data from the published literature, we found seven cases (45–51) of BP associated with IL-12/IL-23 or IL-23 inhibitors (**Table 4**).

**Table 4:** Clinical characteristics of BP cases associated with IL-12/IL-23, IL-23, or IL-17 inhibitors.

Ref	Age/sex	Primary diagnosis	Past history	Biologics	The total dose (mg)	Time tobulla (weeks)	BP occurring site	Anti-BP180/BP230	Serum IgE	BP therapy	Outcome
(46)	75/F	Plaque psoriasis	**ND**	Ustekinumab	225	41	Trunk and extremities	Negative	1,906 U/ml	Methylprednisolone, clobetasol, dexchlorpheniramine, and zinc sulfate (unresponsive). Thus, dapsone was added.	CR
(47)	76/F	Psoriasis	Asthma, osteoporosis, and metabolic syndrome	Ustekinumab	ND	8	Trunk, extremities, head and soles	Negative	1,906 U/ml	Oral corticosteroids	CR
(48)	58/M	Psoriasis	Renal failure, hypertension, and hepatosteatosis	Ustekinumab	90	4	Axillary fossa and inguinal region, trunk and extremities	ND	ND	Topical mometasone furoate, clobetasol propionate	CR
(49)	58/M	Plaque psoriasis	Type 2 diabetes and thyroidectomy	Ustekinumab	90	4	Palms, soles, upper trunk and limbs	ND	ND	Systemic prednisolone and cyclosporine	CR
(50)	62/M	Plaque psoriasis	Hypertension and type 2 diabetes	Ustekinumab	225	40	Upper thighs, lower neck, and upper trunk	ND	ND	Topical corticosteroids	CR
(51)	63/M	Psoriatic onycho-pachydermo periostitis, pustular psoriasis	ND	Ustekinumab	360	76	Trunk and extremities	Anti-BP180: 32.9 U/ml	ND	Oral prednisolone	CR
(45)	76/M	Psoriasis	Diabetes mellitus, chronic heart failure, and atrial fibrillation	Guselkumab	ND	4	Lower limbs	Positive for both	ND	Oral corticosteroid	CR
(52)	65/F	Psoriasis	ND	Secukinumab	150	8 days	Upper limbs and the trunk	Positive but not specific	ND	Topical corticosteroids	CR

BP, bullous pemphigoid; IL, interleukin; Ref, reference; CR, complete remission; ND, not described; F, female; M, male.

#### Demographic characteristics

There were two women (28.6%) and five men (71.4%) with a median age of 63 (mean = 66; range 58–76) years. Guselkumab, an antibody inhibiting IL-23p19, was introduced in one case (14.3%) and ustekinumab, an antagonist of the p40 subunit of IL-12 and IL-23, in the remaining six patients (85.7%) for psoriasis treatment (**Table 2**). Anti-IL-12/IL-23 or IL-23 agents were administered at standard doses and induced blistering lesions during the treatment. The median time to bulla was 8 weeks (mean = 25 weeks; range 4–76 weeks). The trunks and extremities were the most frequently involved sites. Oral mucosal involvement was noticed in one subject (14.3%).

#### Laboratory examination

Histopathologic examinations in all cases showed a subepidermal blister (100%), with eosinophil infiltration in seven cases (100%, 7/7) and neutrophil and lymphocyte infiltration in one case (14.3%, 1/7), respectively.

DIF was performed in all cases, of which seven (100%, 7/7) cases were positive for IgG and C3, two (28.6%, 2/7) for IgM, one (14.3%, 1/7) for C4 and IgA linear deposits along the BMZ, respectively. IIF was documented as positive in five cases (71.4%, 5/7).

Surprisingly, autoantibodies were detected as positive in only two cases (28.6%, 2/7), for anti-BP180NC16a in one and both anti-BP180 and anti-BP230 antibodies in the other one. Eosinophilia and increased serum IgE were observed in two cases (28.6%, 2/7), respectively.

#### Therapy and outcome

All seven patients were given corticosteroids orally and/or topically. Systemic corticosteroids were applied as monotherapy in three cases and with a combination of cyclosporine in another. Systemic and topical steroids were administered in only one case, and dapsone was added to the regimen due to the unsuccessful control (46). Topical corticosteroid monotherapy was prescribed in the remaining two cases. Remission was achieved with the medications above, and no recurrences were observed in the seven patients in the follow-up.

### IL-12/IL-23 or IL-23 inhibitors treating BP

From our knowledge, there was just one case illustrating the therapeutic use of IL-12/IL-23 inhibitors in treating typical BP (27) (**Tables 2**, **3**). In this case, the patient with a long history of psoriasis preceding BP was given ustekinumab due to the recurrence and persistent clinical activity of both diseases, and improvements were rapidly achieved clinically and serologically. Also, cases of anti-laminin-1 pemphigoid that had difficulty in corticosteroid tapering (34) and refractory lichen planus pemphigoids (53, 54) that were successfully treated by IL-12/IL-23 blockers had been reported.

### Pathogenesis of IL-12/IL-23 or IL-23 inhibitors inducing or treating BP

IL-23 is associated with the terminal differentiation of Th17 cells, the primary IL-17-producing cells in BP (55). The biological cascade of IL-17 is performed by an elevated production of a series of inflammatory molecules, including TNF-α (43, 55). Thus, IL-17 and IL-12/IL-23 or IL-23 inhibitors may share a similar mechanism to trigger BP with TNF-α blockers, modifying the immune response of Th1/Th2 and disinhibiting the autoreactive B cells, with TNF-α being a downstream proinflammatory cytokine (56). Additionally, there is such a cycle that dendritic cells (DCs) can secrete IL-23, and TNF-α, as an upstream product of IL-23, can also act on DCs, which makes the regulatory relationship among IL-23, IL-17, and TNF-α a small network (57) (**Figure 1**). Beyond the Th1/Th2 paradigm, other pathways for IL-17 and IL-12/IL-23 or IL-23 inhibitors are involved in the onset of BP. To our knowledge, there was only one case of IL-17 inhibitors inducing BP, and the author postulated that the preexisting low titer of anti-BP180 antibodies, secukinumab-related eosinophil activation, and increased level of Th17 cells during the initial secukinumab administration might contribute to the formation of BP (52). For IL-12/IL-23 inhibitors, however, it was also reported that the balance of Th1/Th2 was not altered during ustekinumab treatment, which indicates that there might be other unknown pathways independent of TNF-α (58, 59). Noteworthy, all of the reported BP cases induced by IL-12/IL-23 or IL-23 inhibitors had a previous history of aTNF-α treatment, increasing susceptibility to BP.

It was reported that there were increased levels of IL-17 and IL-23 in the lesional skin and serum of BP patients (60, 61). IL-17 can upregulate matrix metalloproteinase (MMP)-9 and neutrophil elastase expression from neutrophils, which were involved in the blistering formation and significant in the extension of the disease (60, 62). In addition to being an upstream regulator of IL-17, IL-23 alone can also enhance MMP-9 levels from monocytes (60) (**Figure 1**). That may explain the paradoxical phenomenon of why there were cases of BP successfully treated by IL-17 and IL-12/IL-23 blockers. Except for what is mentioned above, these biological agents may improve the manifestations of BP by controlling its triggers, as psoriasis in our study. However, the mechanism is not yet understood, and further investigations are warranted.

## Interleukin-17 inhibitors

### IL-17 inhibitors inducing BP

IL-17 inhibitors, including secukinumab, brodalumab, and ixekizumab, have been approved for treating moderate-to-severe psoriasis and assessed for non-psoriatic uses, such as hidradenitis suppurativa and alopecia areata (63). Likewise, adverse effects began to manifest, like AIBDs. There were reports about pemphigus caused by secukinumab and brodalumab. But for BP, we found only one case caused by IL-17 inhibitor administration (52).

A 65-year-old woman receiving two doses of 150 mg of secukinumab weekly for psoriasis developed bullae on the upper limbs and trunk 1 day after the second dose. Histological examination was consistent with BP. DIF showed linear deposits of IgG and C3 at the BMZ and a mixed deposition pattern (roof and floor) of both IgG and C3 on salt-split skin immunofluorescence. The serum autoantibodies of BP were found positive, but the type of antibody was not specified. The diagnosis of BP was made, and topical corticosteroids were initiated in addition to the cessation of secukinumab. The BP improved rapidly and was kept in remission even after the reintroduction of secukinumab (**Tables 2**, **4**).

Except for the case described above, secukinumab-caused BP was documented as an adverse effect in some clinical trials but not detailed (64, 65).

### IL-17 inhibitors treating BP

In contrast with inducing BP, IL-17 antagonists have been reported more frequently in treating BP. To our knowledge, the therapeutic use of IL-17 inhibitors for BP coexisting with psoriasis has been described in three cases (**Table 3**), one by ixekizumab (28) and the remaining two by secukinumab (**Table 2**) (29, 30).

Lu et al. (28) reported that a patient with a 20-year history of psoriasis concurrent with BP was treated with ixekizumab at a standard dose for psoriasis due to the patient’s rejection of methotrexate. Clinical remissions of both diseases were rapidly achieved within 2 weeks and still in remission after 1 year of initiating the drug (28).

Secukinumab was applied as an adjuvant therapy combined with oral and topical corticosteroids in another patient and showed a promising efficacy. But the therapeutic effect of secukinumab for BP was not particularly specific due to the combination (30). Yun et al. (29) recently reported the first case describing the clinical improvement of autoantibody-negative BP coexisting with refractory psoriasis treated with secukinumab. Both were still in remission at follow-up, and unexpected better response for BP (29).

Interestingly, one patient was given secukinumab for psoriasis resulting in an original rising of anti-BP180 autoantibody to a peak and decline soon. No clinical improvement of BP was demonstrated, as the patient had no blisters prior to and during the whole duration of secukinumab administration. But as a gradual increase of anti-BP180 antibodies without blister formation was common in patients with relapses, we speculate that secukinumab prevented the recurrence of BP, indicating its underlying therapeutic use (66).

### Pathogenesis of IL-17 inhibitors inducing or treating BP

As aforementioned in the IL-12/IL-23 inhibitor section, IL-17 inhibitors can cause BP by regulating TNF-α, thereby modulating its downstream pathways. There might be unknown pathways apart from T-cell imbalance, and the mechanism remains to be elucidated (56). In addition to Th17 cells, the main sources of IL-17, recruited inflammatory cells including eosinophils and mast cells can also produce IL-17 (38). IL-17 can increase MMP-9 production from eosinophils and neutrophil elastase release from neutrophils, which were associated with the degrading of BP180 and the formation of the subepidermal cleft, thus blocking it could have a therapeutic effect (62). In the meantime, neutrophils affected by IL-17 can, in turn, promote IL-17 production, turning into a cascade loop (67) (**Figure 1**).

## Other biologics inducing or treating BP

In addition, some biologics can only cause or treat BP. In terms of inducing BP, immune checkpoint inhibitors targeting PD-1/PD-L1, which are used to treat advanced solid malignancies, can cause immune-related adverse effects including BP. There were many cases and reviews relevant to BP cases caused by PD-1/PD-L1 inhibitors including pembrolizumab, nivolumab, and cemiplimab, but no reports about treating BP to date. Nonspecific activation of the immunity and the shared antigens between tumor cells and the BMZ have been involved in the development of PD-1/PD-L1 blockers-inducing BP (3, 4, 68–70). In the treatment of BP, omalizumab, an antibody targeting IgE, and dupilumab, an antibody binding IL-4 receptor subunit α (IL-4Rα), are now increasingly utilized for the treatment of BP as novel therapeutic approaches (71–73).

## Conclusion and further perspectives

With the broader indications and more extensive clinical practice of biologics, the incidence of adverse effects like BP will be much more common. As the early manifestations of drug-induced BP are not specific, biologics-induced BP might be underreported, and diagnoses are challenging. Dermatologists should keep this rare but life-threatening adverse effect in mind and be more cautious on identifying the disease and initiating treatment as early as possible.

Reviewing all of the 25 cases of BP induced by biological agents collected in our study, we would like to know if there are some common threads for the patients who developed BP. Although the number of cases was limited and the recorded data were uneven, we can still distill out some possible common threads. Patients are usually younger. The average age of all 25 patients was 60 years, which is younger than that of spontaneous BP (74). And in our recorded cases, the positive rate of anti-BP230 was low, with only one out of 25 cases being positive (**Tables 1**, **4**).

The findings raised above may not have a high degree of confidence, but they provide us with new perspectives and future research directions that may indicate underlying mechanisms other than those mentioned in this article.

In addition to the direct effects of those culprit drugs, the primary diseases and polypharmacy in patients may act as predisposing factors and have an underlying relationship with BP occurrence (75). Patients involved in our study were usually elderly and fragile and had a long history of psoriasis and other diseases. Psoriasis has been associated with a higher incidence of BP. It is hypothesized that epigenetic changes altered by psoriasis lesions may trigger or accelerate autoreactive response to specific antigens, resulting in autoantibody production to induce BP. Neutrophil elastase may also be involved in subepidermal blister formation (76). For other involved diseases treated with biologics, there are also reports about epitope spreading, such as the plectin hypothesis, which indicates that the inflammation present in inflammatory bowel disease exposes plectin within the bowel. Plectin exposures can stimulate the production of autoantibodies to cross-react with the skin of certain susceptible individuals, provoking a secondary immune response resulting in BP occurrence (77).

We also tried to refine and summarize the features of a particular type of BP patient responding to biologics, which may have significant clinical implications. Noteworthy, six of the seven cases were comorbid with psoriasis, and multiple medications for psoriasis were prescribed, indicating that those biologics might be effective in treating BP concomitant with psoriasis. We can also assume that they can also be used for treating BP concurrent with other immune diseases except for psoriasis. To our knowledge, etanercept is the most commonly used aTNF-α in the treatment of BP (**Table 2**), and clinical trials evaluating the effect of IL-17 inhibitors on BP are ongoing as well (78). Interestingly, a case of etanercept-induced BP improved after switching to adalimumab (22), suggesting that biologics with different preparation processes may have a distinct effect even focusing on the same target.

The optimal therapeutic recommendations for biologics-induced BP have yet to be well-established (78), and questions about the mechanism of the paradoxical phenomena that these agents can both cause and treat BP still need to be fully elucidated. Further investigations concerning these issues are warranted to improve our understanding of this phenomenon, and we believe that it would be significant for future optimized treatments of BP.

## Author contributions

The manuscript was written by JZ and S-HW with significant contributions from Y-GZ. All authors contributed to the article and approved the submitted version.
